# Progress towards the UNAIDS 90‐90‐90 targets among persons aged 50 and older living with HIV in 13 African countries

**DOI:** 10.1002/jia2.26005

**Published:** 2022-09-29

**Authors:** Shannon M. Farley, Chunhui Wang, Rachel M. Bray, Andrea Jane Low, Stephen Delgado, David Hoos, Angela N. Kakishozi, Tiffany G. Harris, Rose Nyirenda, Nellie Wadonda, Michelle Li, Mbaraka Amuri, James Juma, Nzali Kancheya, Ismela Pietersen, Nicholus Mutenda, Salomo Natanael, Appolonia Aoko, Evelyn W. Ngugi, Fred Asiimwe, Shirley Lecher, Jennifer Ward, Prisca Chikwanda, Owen Mugurungi, Brian Moyo, Peter Nkurunziza, Dorothy Aibo, Andrew Kabala, Sam Biraro, Felix Ndagije, Godfrey Musuka, Clement Ndongmo, Judith Shang, Emily K. Dokubo, Laura E. Dimite, Rachel McCullough‐Sanden, Anne‐Cecile Bissek, Yimam Getaneh, Frehywot Eshetu, Tepa Nkumbula, Lyson Tenthani, Felix R. Kayigamba, Wilford Kirungi, Joshua Musinguzi, Shirish Balachandra, Eugenie Kayirangwa, Ayiyi Ayite, Christine A. West, Stephane Bodika, Katrina Sleeman, Hetal K. Patel, Kristin Brown, Andrew C. Voetsch, Wafaa M. El‐Sadr, Jessica J. Justman

**Affiliations:** ^1^ ICAP at Columbia University New York City New York USA; ^2^ Department of Epidemiology Mailman School of Public Health Columbia University New York City New York USA; ^3^ Malawi Ministry of Health Lilongwe Malawi; ^4^ US Centers for Disease Control and Prevention (CDC) Lilongwe Malawi; ^5^ CDC Mbabane Eswatini; ^6^ CDC Dar es Salaam Tanzania; ^7^ Ministry of Health Community Development Gender, Elderly and Children through The National AIDS Control Program (NACP) Dodoma Tanzania; ^8^ CDC Lusaka Zambia; ^9^ CDC Windhoek Namibia; ^10^ Ministry of Health and Social Services Windhoek Namibia; ^11^ CDC Nairobi Kenya; ^12^ CDC Maseru Lesotho; ^13^ CDC Kampala Uganda; ^14^ CDC Harare Zimbabwe; ^15^ Zimbabwe Ministry of Health and Child Care Hararre Zimbabwe; ^16^ CDC Yaounde Cameroon; ^17^ Cameroon Ministry of Public Health Yaounde Cameroon; ^18^ Ethiopia Public Health Institute Addis Ababa Ethiopia; ^19^ CDC Addis Ababa Ethiopia; ^20^ Uganda Ministry of Health Kampala Uganda; ^21^ CDC Abidjan Cote d'Ivoire; ^22^ CDC Kigali Rwanda; ^23^ CDC Atlanta Georgia USA

**Keywords:** ageing, HIV epidemiology, HIV testing, older PLHIV, PHIA, UNAIDS goals

## Abstract

**Introduction:**

Achieving optimal HIV outcomes, as measured by global 90‐90‐90 targets, that is awareness of HIV‐positive status, receipt of antiretroviral (ARV) therapy among aware and viral load (VL) suppression among those on ARVs, respectively, is critical. However, few data from sub‐Saharan Africa (SSA) are available on older people (50+) living with HIV (OPLWH). We examined 90‐90‐90 progress by age, 15–49 (as a comparison) and 50+ years, with further analyses among 50+ (55–59, 60–64, 65+ vs. 50–54), in 13 countries (Cameroon, Cote d'Ivoire, Eswatini, Ethiopia, Kenya, Lesotho, Malawi, Namibia, Rwanda, Tanzania, Uganda, Zambia and Zimbabwe).

**Methods:**

Using data from nationally representative Population‐based HIV Impact Assessments, conducted between 2015and 2019, participants from randomly selected households provided demographic and clinical information and whole blood specimens for HIV serology, VL and ARV testing. Survey weighted outcomes were estimated for 90‐90‐90 targets. Country‐specific Poisson regression models examined 90‐90‐90 variation among OPLWH age strata.

**Results:**

Analyses included 24,826 HIV‐positive individuals (15–49 years: 20,170; 50+ years: 4656). The first, second and third 90 outcomes were achieved in 1, 10 and 5 countries, respectively, by those aged 15–49, while OPLWH achieved outcomes in 3, 13 and 12 countries, respectively. Among those aged 15–49, women were more likely to achieve 90‐90‐90 targets than men; however, among OPLWH, men were more likely to achieve first and third 90 targets than women, with second 90 achievement being equivalent. Country‐specific 90‐90‐90 regression models among OPLWH demonstrated minimal variation by age stratum across 13 countries. Among OLPWH, no first 90 target differences were noted by age strata; three countries varied in the second 90 by older age strata but not in a consistent direction; one country showed higher achievement of the third 90 in an older age stratum.

**Conclusions:**

While OPLWH in these 13 countries were slightly more likely than younger people to be aware of their HIV‐positive status (first 90), this target was not achieved in most countries. However, OPLWH achieved treatment (second 90) and VL suppression (third 90) targets in more countries than PLWH <50. Findings support expanded HIV testing, prevention and treatment services to meet ongoing OPLWH health needs in SSA.

## 1 INTRODUCTION

Persons living with HIV (PLWH), including those in low‐ and middle‐income countries, have experienced extended life expectancies due to the success of antiretroviral (ARV) therapies [1]. In 2014, the Joint United Nations Programme on HIV and AIDS (UNAIDS) launched the 90‐90‐90 global targets for 2020: 90% of PLWH will know their HIV status (first 90); 90% of those aware will receive sustained ARV therapy (ART) (second 90); and of these, 90% will achieve viral load suppression (VLS) (third 90) [2], as a stepping stone towards achieving the end of the AIDS epidemic as a public health threat by 2030 with 95‐95‐95 global targets for 2025 [3, 4]. In 2019, a modelling study examined data released by UNAIDS and found that 60 out of 170 countries were able to report on all three 90 targets; many of these countries, however, were not likely to achieve 90‐90‐90 targets by 2020 although three countries in sub‐Saharan Africa (SSA) were among the six that achieved the model targets [5]. Studies of HIV prevalence among older adults, 50 years of age and older (50+), have shown increases in the number of older persons living with HIV (OPLWH), in general, and particularly in Eastern and Southern Africa [1, 6, 7].

With this growing and ageing population of OPLWH, it is important to focus on older adults and their risk for HIV as well as to understand how OPLWH have progressed towards the UNAIDS 90‐90‐90 targets in SSA [8]. A recent systematic review and meta‐analysis using data published between 2014 and 2018 among PLWH aged 15 and older that examined progress towards 90‐90‐90 targets by a range of socio‐demographics found a mixed picture in SSA [9]. Both males and females have made progress towards achieving the 90‐90‐90 targets by age group. Women within each age group demonstrated more progress along the cascade overall compared with men, and older adults achieved more progress towards the 90‐90‐90 targets than younger age groups [9].

To date, measuring progress towards 90‐90‐90 among the OPLWH has been challenging for several reasons. First, studies examining HIV in SSA have largely focused on younger adults, despite the growth in the number of OPLWH [1, 6, 10, 11]. Secondly, not all studies have reported on all three 90s, usually focusing on one or two of the 90s, with more recent attempts examining all 90‐90‐90 targets [5, 12–15]. Thirdly, most studies that assessed 90‐90‐90 progress have relied on programmatic data, which are restricted to the subset of the population living with HIV who have accessed services, rather than the entirety of people living with HIV [16].

We examined 90‐90‐90 target achievement by age and sex using nationally representative samples of adults in 13 SSA countries: Cameroon, Cote d'Ivoire, Eswatini, Ethiopia, Kenya, Lesotho, Malawi, Namibia, Rwanda, Tanzania, Uganda, Zambia and Zimbabwe. We further assessed the progress towards 90‐90‐90 targets among subsets of individuals by age strata among OPLWH. Finally, we assessed the associations between age and achievement of the 90‐90‐90 targets among OPLWH, by country, using Poisson regression models, to inform efforts to address gaps in services for this population.

## 2 METHODS

### 2.1 Data source

Data for the 13 countries (Cameroon 2017–2018, Cote d'Ivoire 2017–2018, Eswatini 2016, Ethiopia 2017–2018, Kenya 2018, Lesotho 2016–2017, Malawi 2015–2016, Namibia 2017, Rwanda 2018–2019, Tanzania 2016–2017, Uganda 2016–2017, Zambia 2016 and Zimbabwe 2015–2016) were collected as part of the Population‐based HIV Impact Assessment (PHIA) surveys between 2015 and 2019. The PHIA surveys selected a nationally representative sample using a stratified two‐stage cluster sampling design to provide a population‐level assessment of the burden of HIV at national and sub‐national levels [17, 18]. The surveys were funded by the United States President's Emergency Plan for AIDS Relief (PEPFAR) and conducted by ministries of health with support from ICAP at Columbia University, the University of California at San Francisco (Namibia PHIA) and the US Centers for Disease Control and Prevention (CDC). The PHIA survey design and implementation have been previously described [18, 19]. At the time the PHIAs were conceived, the HIV burden varied across these countries among 15–49 year old; seven countries had an HIV prevalence ranging from 1.5% to 7.3% (Ethiopia—1.5%, Rwanda—3.0%, Cote d'Ivoire—3.7%, Cameroon—4.3%, Tanzania—5.3%, Kenya—5.6% and Uganda—7.3%); four countries had an HIV prevalence ranging from 10.6% to 15.0% (Malawi—10.6%, Namibia—14.3%, Zambia—14.3% and Zimbabwe—15.0%); and two countries had an HIV prevalence above 20% (Lesotho—23% and Eswatini—26%). The ARV coverage varied among these countries at that time as well; six countries had ARV coverage below 50% (Cote d'Ivoire—24.4%, Cameroon—26.0%, Lesotho—35.0%, Tanzania—37.0%, Ethiopia—40.0% and Uganda—40.0%); and seven countries had ARV coverage well above 50% (Zambia—76.9%, Kenya—78.5%, Eswatini—82.0%, Malawi—83.0%, Namibia—90.0%, Zambia—90.0% and Rwanda—91.0%) [20, 21, 22, 23, 24, 25, 26, 27, 28, 29, 30, 31, 32].

### 2.2 Eligibility criteria and survey domains

Eligibility criteria for this analysis were based on age ≥15 years old and whether the person slept in the household the night before the interview. Upper age limits for individual eligibility varied by country: 59 years of age for Lesotho and Zambia, 64 years of age for Cameroon, Cote d'Ivoire, Ethiopia, Kenya, Malawi, Namibia, Rwanda and Uganda, and no upper age limit in Eswatini, Tanzania and Zimbabwe. Household and individual interviews were conducted among consenting individuals (minors provided informed assent), capturing demographic, behavioural and clinical data, including self‐reported HIV status, testing history and medication uptake.

### 2.3 Laboratory procedures

Consenting participants provided blood specimens for HIV diagnostic testing, using the national HIV rapid testing algorithm and counselling was provided to all survey participants in their homes. Blood specimens for all HIV‐positive participants were further tested for CD4^+^ T‐cell enumeration in the household, viral load (VL) testing at central laboratories and qualitative testing for selected ARVs, at the University of Cape Town, South Africa. VL testing was performed on the Abbott *m*2000 (Abbott Molecular, Des Plaines, IL, USA), the bioMérieux NucliSens EasyMag/EasyQ (bioMérieux, Marcy‐l’Étoile, France) or the Roche COBAS AmpliPrep/COBAS TaqMan (Roche Diagnostics, Pleasanton, CA, USA) platforms. VLS was defined as <1000 copies/ml [33].

### 2.4 Ethics approval

The surveys were approved by the Institutional Review Boards at Columbia University Irving Medical Center, the University of California at San Francisco (Namibia only), the CDC and the local ethics board in each country.

### 2.5 Measures

We examined 90‐90‐90 progress by sex and age (15–49, 50+) in the 13 PHIAs. The first 90 (HIV status awareness) outcome was defined as the proportion of participants who tested HIV positive during the PHIAs, who self‐reported being HIV positive during the interview, prior to learning the result of household HIV testing, or if ARVs were detected in their blood. The second 90 (ART use among aware) was defined as the proportion of aware participants who self‐reported taking ARVs or had ARVs detected in their blood, and the third 90 (VLS) was defined as the proportion of ART users who have HIV‐1 RNA <1000 copies/ml.

Descriptive statistics and model variables included basic demographics, such as sex, age groups (50–54, 55–59, 60–64, 65 and older), residence and other measures of socio‐economic status as well as a variety of treatment outcome variables.

### 2.6 Analysis

We calculated 90‐90‐90 estimates by age (15–49 and 50+) and sex for each of the 13 countries, and accounted for survey design with Jackknife variance estimation. These analyses were performed with SAS version 9.4. We calculated weighted percentages to describe demographic characteristics, partner's HIV status, time since HIV diagnosis and time on ART, among OPLWH from all 13 countries, using the Taylor Series method on variance estimation. We pooled the variance strata and Primary Sampling Units recorded on the country‐level data files, re‐numbered strata to make unique ones, then produced the variance estimates with the Taylor Series linearization method in the pooled dataset. We fit three multivariate Poisson regression models per country, one for each of the 90‐90‐90 targets, among OPLWH, to examine how 90‐90‐90 progress varied by age strata among OPLWH (50–54, 55–59, 60–64, 65 and older) [34, 35]. We adjusted the first 90 models for sex, residence, employed, wealth quintile, education, marital status and partner HIV status‐self‐reported. We adjusted the second 90 models for the same variables as the first 90 models as well as the number of years since diagnosis. We adjusted the third 90 models for the same variables as the first 90 and the number of years since initiating ART. We reported adjusted prevalence ratios (aPR) to measure the association between these factors and the 90‐90‐90 outcomes. We constructed models using Stata 15. All comparisons reported are significant at *p* < 0.05.

## 3 RESULTS

### 3.1 90‐90‐90 targets

**Figure 1:**
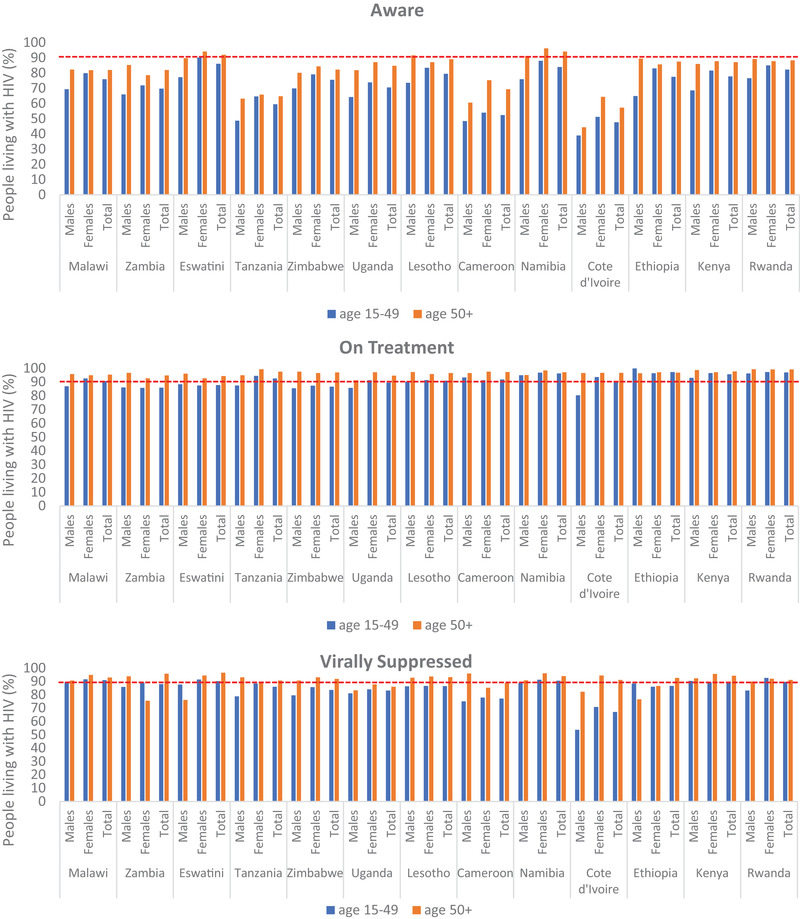
Antiretroviral‐adjusted 90‐90‐90 estimates among 15–49 and 50+ years, 13 countries, Population‐based HIV Impact Assessments, 2015–2019. Notes: – – – – – – represents the 90‐90‐90 target; Survey dates: Cameroon 2017–2018, Cote d'Ivoire 2017–2018, Eswatini 2016, Ethiopia 2017–2018, Kenya 2018, Lesotho 2016–2017, Malawi 2015–2016, Namibia 2017, Rwanda 2018–2019, Tanzania 2016–2017, Uganda 2016–2017, Zambia 2016 and Zimbabwe 2015–2016.

A total of 20,170 PLWH 15–49 years of age and 4656 OPLWH 50+ were included in the analyses. Figure 1 shows the progress towards 90‐90‐90 targets for each of the 13 countries. The target for awareness of HIV‐positive status (first 90) was achieved among women aged 15–49 and among 50+ (women and men) in Eswatini, among 50+ (women and men) in Namibia, as well as among men 50+ in Lesotho. The target for ARV treatment (second 90) was achieved among those aged 15–49 (women and men) in six countries and among women aged 15–49 in four other countries, while the target was achieved in all 13 countries among 50+ (women and men). The VLS target (third 90) was achieved by those aged 15–49 (women and men) in one country and by women aged 15–49 in three countries and men aged 15–49 in one country, while the VLS target was achieved among 50+ (women and men) in eight countries and men 50+ in three countries and women 50+ in one country. Among those aged 15–49, women were more likely to have achieved the 90‐90‐90 targets than men; however among those 50+, men were more likely to have achieved the first (3 countries vs. 2 countries, respectively) and third (11 countries vs. 9 countries, respectively) 90 targets than women, with second 90 achievement being equivalent.

### 3.2 OPLWH demographics

Among all 4656 OPLWH from the 13 countries, as noted in Table 1, there were more women than men (56.42% vs. 43.68%) identified, with the majority of OPLWH in the 50–59 age range (50–54 years: 46.27%; 55–59 years: 32.55%), the majority resided in rural areas (60.89%) and were not currently employed (57.23%). The OPLWH had a similar wealth status as the general population, with 61.65% in the upper 60% wealth quintile for their country. Most men (78.71%) were married or living with a partner compared with just 26.69% of women, while almost half of women (47.27%) were widowed compared with just 8.60% of men. The highest level of educational completion for most (58.37%) was a primary school. Most (79.44%) of the women reported no partner in the household compared with 31.75% of men, while 39.40% of the men reported having one or more HIV‐positive partners compared to 8.20% of the women. Among OPLWH, 66.09% of the women reported having no sexual partners in the past 12 months compared with 20.04% of men, and 36.05% of the men reported that one or more of their partners think/told/tested HIV positive compared with 10.44% of the women.

**Table 1:** Pooled 13 country descriptive socio‐demographic statistics among older persons living with HIV aged 50 and over

	Males		Females		Total	
	*N*	% (95% CIs)	*N*	% (95% CIs)	*N*	% (95% CIs)
Sex						
Male					1911	43.58 (42.15–45.02)
Female					2745	56.42 (54.98–57.85)
Age						
50–54	837	46.51 (44.01–49.01)	1275	46.08 (44.07–48.09)	2112	46.27 (44.73–47.81)
55–59	606	32.73 (30.52–34.94)	900	32.42 (30.64–34.19)	1506	32.55 (31.11–34.00)
60–64^a^	335	15.87 (14.45–17.3)	437	16.83 (15.38–18.27)	772	16.41 (15.41–17.42)
65+^b^	133	4.89 (4.08–5.69)	133	4.67 (3.67–5.67)	266	4.77 (4.08–5.45)
Residence						
Urban	640	38.21 (36.11–40.30)	939	39.81 (37.93–41.68)	1579	39.11 (37.72–40.50)
Rural	1271	61.79 (59.70–63.89)	1806	60.19 (58.32–62.07)	3077	60.89 (59.50–62.28)
Employed						
Yes	907	54.30 (52.06–56.54)	799	33.85 (31.99–35.72)	1706	42.77 (41.32–44.22)
No	1004	45.70 (43.46–47.94)	1943	66.15 (64.28–68.01)	2947	57.23 (55.78–58.68)
Wealth quintile						
Lower 40%	802	35.95 (33.87–38.02)	1288	40.20 (38.41–41.99)	2090	38.35 (37.00–39.69)
Upper 60%	1108	64.05 (61.98–66.13)	1455	59.80 (58.01–61.59)	2563	61.65 (60.31–63.00)
Marital status						
Never married	102	2.98 (2.35–3.61)	276	6.28 (5.27–7.30)	378	4.84 (4.24–5.45)
Married or living together	1449	78.71 (76.86–80.56)	764	26.69 (24.93–28.45)	2213	49.36 (47.65–51.07)
Divorced or separated	161	9.70 (8.26–11.14)	389	19.76 (17.96–21.56)	550	15.38 (14.05–16.71)
Widowed	196	8.60 (7.35–9.85)	1310	47.27 (45.33–49.21)	1506	30.42 (29.02–31.81)
Education						
None	246	10.45 (9.03–11.87)	551	26.02 (24.03–28.00)	797	19.22 (17.88–20.55)
Primary	1055	59.36 (57.20–61.52)	1589	57.61 (55.62–59.59)	2644	58.37 (56.79–59.96)
Post primary	605	30.18 (28.16–32.21)	592	16.38 (15.18–17.57)	1197	22.41 (21.28–23.53)
Partner HIV status‐measured						
One or more partner HIV positive	749	39.40 (37.27–41.53)	265	8.20 (7.22–9.18)	1014	21.80 (20.41–23.19)
All partners HIV positive	393	23.96 (21.96–25.96)	128	5.37 (4.53–6.20)	521	13.47 (12.48–14.45)
Don't know or missing	82	4.89 (3.88–5.89)	191	6.99 (6.25–7.74)	273	6.08 (5.47–6.68)
No partner in household	687	31.75 (29.65–33.86)	2161	79.44 (77.97–80.90)	2848	58.65 (57.00–60.31)
Partner HIV status‐self‐reported						
One or more partner think/told/tested HIV positive	736	36.05 (33.84–38.26)	387	10.44 (9.52–11.35)	1123	21.52 (20.25–22.78)
All partners think/told/tested HIV negative	359	20.47 (18.75–22.19)	207	8.05 (6.99–9.10)	566	13.42 (12.43–14.41)
Don't know or missing	433	23.44 (21.45–25.42)	455	15.43 (13.93–16.92)	888	18.89 (17.58–20.21)
No partner in the past 12 months	370	20.04 (17.94–22.14)	1692	66.09 (64.09–68.09)	2062	46.17 (44.29–48.05)
Country						
Malawi	155	9.16 (8.11–10.21)	184	8.27 (7.44–9.10)	339	8.66 (8.15–9.17)
Zambia	148	8.79 (7.97–9.61)	184	6.74 (6.03–7.46)	332	7.64 (7.13–8.14)
Eswatini	243	2.16 (1.91–2.41)	337	1.69 (1.52–1.86)	580	1.90 (1.76–2.04)
Tanzania	149	17.76 (15.62–19.90)	225	20.51 (18.84–22.18)	374	19.31 (18.29–20.33)
Zimbabwe	348	16.00 (14.46–17.55)	428	11.95 (11.09–12.81)	776	13.72 (12.91–14.53)
Uganda	116	10.66 (9.73–11.59)	167	10.34 (9.25–11.44)	283	10.48 (9.76–11.20)
Lesotho	209	3.03 (2.65–3.41)	339	2.84 (2.60–3.09)	548	2.92 (2.72–3.12)
Cameroon	75	5.4 (4.67–6.13)	113	6.28 (5.56–7.00)	188	5.90 (5.35–6.45)
Namibia	183	2.12 (1.85–2.38)	311	2.34 (2.14–2.53)	494	2.24 (2.10–2.38)
Cote d'Ivoire	42	4.44 (3.68–5.19)	65	6.26 (5.12–7.39)	107	5.46 (4.79–6.13)
Ethiopia	33	3.72 (3.12–4.32)	52	3.22 (2.88–3.57)	85	3.44 (3.13–3.75)
Kenya	121	13.49 (12.10–14.88)	203	16.19 (14.96–17.42)	324	15.02 (14.23–15.80)
Rwanda	89	3.27 (2.90–3.65)	137	3.36 (3.04–3.68)	226	3.32 (3.14–3.51)

Note: Upper age limits varied by country.

^a^ Excludes Lesotho and Zambia.

^b^ Includes only Eswatini, Tanzania and Zimbabwe.

On residence, Lesotho had three categories: urban, rural and peri‐urban, and peri‐urban was collapsed into urban, while in Ethiopia, small urban was classified as rural and large urban was classified as urban.

### 3.3 OPLWH treatment status

Among all OPLWH in Table 2, 21.25% were unaware of their HIV‐positive status. Among OPLWH, 27.21% did not have ARVs detected in their blood and 26.50% were not virally suppressed. More than one‐third (37.04%) of OPLWH had a CD4^+^ cell count ≥500 per μl, 27.03% had a CD4^+^ cell count of 350–499 per μl, 22.71% had a CD4^+^ cell count of 200–349 per μl, 10.87% had a CD4^+^ count of 100–199 per μl and 2.36% had a CD4^+^ count of <100 per μl. Of all OPLWH, 12.68% indicated that they were never tested for HIV before the survey, and among those who were aware of their HIV‐positive status, 6.82% had been diagnosed within the past year. Almost all OPLWH (96.99%) with detectable ARVs in their blood were taking first‐line ART regimens (guidance at the time of the surveys) and more than half (54.84%) of those on ART indicated that they initiated treatment 5 or more years prior to the survey.

**Table 2:** Pooled 13 country descriptive clinical statistics among older persons living with HIV aged 50 and over

	Males		Females		Total	
	*N*	% (95% CIs)	*N*	% (95% CIs)	*N*	% (95% CIs)
Diagnosis and treatment status (ARV‐adj)						
Unaware of HIV status	316	22.46 (20.16–24.76)	404	20.31 (18.72–21.89)	720	21.25 (19.94–22.56)
Aware of HIV status and not on ART	60	2.98 (2.36–3.60)	94	2.49 (1.98–3.00)	154	2.70 (2.30–3.11)
Aware of HIV status and on ART	1535	74.56 (72.20–76.91)	2247	77.20 (75.56–78.84)	3782	76.05 (74.71–77.39)
ARVs detected						
Yes	1467	70.39 (67.99–72.79)	2165	74.65 (72.92–76.38)	3632	72.79 (71.34–74.23)
No	437	29.61 (27.21–32.01)	566	25.35 (23.62–27.08)	1003	27.21 (25.77–28.66)
VLS						
Yes	1460	70.90 (68.51–73.29)	2176	75.51 (73.74–77.27)	3636	73.50 (72.02–74.97)
No	450	29.10 (26.71–31.49)	566	24.49 (22.73–26.26)	1016	26.50 (25.03–27.98)
CD4 cell count per μl						
<100	50	3.19 (2.58–3.81)	37	1.69 (1.13–2.24)	87	2.36 (1.95–2.76)
100–199	221	14.57 (12.89–16.25)	161	7.89 (6.48–9.31)	382	10.87 (9.77–11.97)
200–349	473	29.09 (26.69–31.49)	414	17.58 (15.79–19.37)	887	22.71 (21.11–24.30)
350–499	453	27.11 (24.88–29.34)	566	26.97 (24.90–29.04)	1019	27.03 (25.32–28.75)
≥500	500	26.04 (23.80–28.27)	1215	45.88 (43.58–48.17)	1715	37.04 (35.32–38.75)
Testing history						
Never tested	155	11.39 (9.49–13.29)	243	13.69 (12.15–15.22)	398	12.68 (11.67–13.70)
Tested in the past year	547	27.83 (25.98–29.68)	719	26.44 (24.75–28.13)	1266	27.04 (25.74–28.34)
Tested more than 1 year ago	1113	56.61 (54.22–59.01)	1663	56.27 (54.39–58.14)	2776	56.42 (54.98–57.86)
Don't know or missing	96	4.17 (3.42–4.93)	120	3.61 (2.85–4.37)	216	3.85 (3.33–4.38)
Number of years since diagnosis						
Less than 12 months	97	6.96 (5.98–8.02)	135	6.71 (5.78–7.64)	232	6.82 (6.11–7.53)
1 to less than 5 years	453	32.32 (29.97–34.66)	634	31.78 (29.84–33.71)	1087	32.01 (30.44–33.58)
5 years or more	868	60.73 (58.30–63.15)	1334	61.51 (59.59–63.43)	2202	61.17 (59.6–62.75)
Antiretroviral regimen (among ARV detected)						
First line (EVP, NVP and INSTI)	1435	96.89 (96.38–97.40)	2120	97.06 (96.30–97.83)	3555	96.99 (96.49–97.49)
Second line (PI, LPV and ATV)	29	3.07 (2.57–3.58)	42	2.71 (1.95–3.47)	71	2.86 (2.36–3.36)
Both	2	0.04 (0.00–0.09)	3	0.23 (0.22–0.23)	5	0.15 (0.13–0.17)
Number of years since initiating ART						
Less than 12 months	125	8.78 (7.77–9.79)	196	10.69 (9.25–12.12)	321	9.87 (8.96–10.78)
1 to less than 5 years	466	35.43 (33.07–37.79)	647	35.18 (33.15–37.22)	1113	35.29 (33.66–36.91)
5 years or more	758	55.79 (53.45–58.13)	1131	54.13 (52.11–56.16)	1889	54.84 (53.28–56.41)

Note: CD4 cell count does not include Kenya or Rwanda as CD4 testing was not conducted.

Abbreviations: ART, antiretroviral therapy; ARV, antiretroviral; VLS, viral load suppression; EVP, Emtricitabine/rilpivirine/tenofovir; NVP, Nevirapine; INSTI, Integrase strand transfer inhibitors; PI, protease inhibitor; LPV, Lopinavir; ATV, Atazanavir.

Definitions: Diagnosis and treatment status: Percent distribution of HIV‐positive persons by HIV diagnosis and treatment status; ARVs detected: Percent distribution of HIV‐positive persons by the presence of detectable ARVs; VLS: Among HIV‐positive persons, percentage with viral load suppression (< 1000 copies/ml); CD4 cell count per μl: Among HIV‐positive persons, percentage with CD4 count within each range; Testing history: Percentage of persons who ever received HIV testing and received their test results; Number of years since diagnosis: Percent distribution of HIV‐positive persons by time since diagnosis; Antiretroviral regimen (among ARV detected): Among those with detected ARVs, percent distribution on first line, second line or both regimens; Number of years since initiating ART: Among those self‐reporting taking ARVs, percent distribution of time since initiating ART.

### 3.4 OPLWH country‐specific Poisson regression

Table 3 shows the first 90 awareness models for all 13 countries. There were no significant differences in awareness of HIV‐positive status by age stratum among OPLWH (*p* > 0.05). In Table 4, the second 90 on treatment models for all 13 countries are presented. Three countries showed variation in the second 90 by older age strata but not in a consistent direction. With regard to the second 90, in Eswatini, those aged 60–64 who were aware of their HIV‐positive status were more likely to be on treatment than those aged 50–54 years old (aPR: 1.06, 95% confidence intervals [CI]: 1.01–1.11, *p* < 0.05). In Cameroon, those aged 60–64 who were aware of their HIV‐positive status were less likely to be on treatment than those 50–54 years old (aPR: 0.95, 95% CIs: 0.91–0.99, *p* < 0.05). In Rwanda, those aged 55–59 who were aware of their HIV‐positive status were more likely to be on treatment than those 50–54 years old (aPR: 1.01, 95% CIs: 1.00–1.02, *p* < 0.05), while those 60–64 years of age were less likely to be on treatment than those 50–54 years old (aPR: 0.98, 95% CIs: 0.96–1.00, *p* < 0.05). Table 5 shows the third 90 VLS models for the 13 countries. One country showed higher achievement of the third 90 in an older age stratum. In Ethiopia, those aged 60–64 who were on treatment were more likely to have VLS compared to those 50–54 years old (aPR: 1.10, 95% CIs: 1.00–1.20, *p* < 0.05).

**Table 3:** Poisson regression models for awareness of HIV‐positive status (first 90), Population‐based HIV Impact Assessments, 13 countries, 2015–2019

	Aged 50–54			Aged 55–59			Aged 60–64			Aged 65+		
Country	*n*	% (95% CI)	aPR^a^	*n*	% (95% CI)	aPR^a^	*n*	% (95% CI)	aPR^a^	*n*	% (95% CI)	aPR^a^
Malawi	133	80.28 (73.57–86.99)	1.00	91	82.32 (74.81–89.83)	1.03 (0.87–1.22)	59	85.78.18–93.06)	1.04 (0.87–1.25)	NA		
Zambia	175	82.80 (79.39–86.22)	1.00	97	80.58 (76.53–84.62)	0.93 (0.84–1.03)	NA			NA		
Eswatini	203	92.05 (88.45–95.65)	1.00	140	92.77 (88.31–97.23)	1.01 (0.95–1.08)	111	91.68 (86.32–97.04)	0.98 (0.91–1.07)	84	90.61 (83.83–97.39)	0.99 (0.89–1.11)
Tanzania	107	69.95 (64.15–75.76)	1.00	77	69.43 (60.30–78.55)	0.96 (0.76–1.21)	43	54.99 (41.33–68.66)	0.83– (0.55–1.25)	30	47.61 (35.47–59.76)	0.73 (0.48–1.12)
Zimbabwe	242	85.33 (80.98–89.69)	1.00	195	81.74 (76.26–87.23)	0.95 (0.86–1.05)	121	82.32 (77.17–87.46)	0.96 (0.87–1.06)	92	75.91 (68.66–83.16)	0.89 (0.76–1.04)
Uganda	115	86.48 (82.72–90.24)	1.00	68	79.75 (73.49–86.00)	0.91 (0.74–1.13)	55	90.02 (85.58–94.46)	1.04 (0.88–1.23)	NA		
Lesotho	263	90.17 (87.22–93.11)	1.00	226	87.76 (84.38–91.13)	0.98 (0.92–1.04)	NA			NA		
Cameroon	55	70.03 (65.54–74.52)	1.00	33	68.39 (53.86–82.92)	0.96 (0.64–1.44)	38	69.30 (62.00–76.60)	0.98 (0.76–1.26)	NA		
Namibia	225	92.88 (89.51–96.24)	1.00	150	95.07 (92.27–97.87)	1.01 (0.95–1.07)	90	96.85 (92.88–100.00)	1.05 (0.97–1.13)	NA		
Cote d'Ivoire	27	70.02 (62.40–77.64)	1.00	22	55.27 (38.82–71.72)	0.86 (0.60–1.24)	11	42.48 (28.64–56.32)	0.61 (0.32–1.15)	NA		
Ethiopia	42	88.97 (84.62–93.32)	1.00	15	87.26 (82.22–92.30)	0.99 (0.78–1.26)	18	83.72 (70.56–96.88)	0.96 (0.70–1.33)	NA		
Kenya	134	87.64 (84.23–91.05)	1.00	87	80.75 (77.00–84.50)	0.91 (0.81–1.02)	62	96.06 (95.43–96.68)	1.07 (0.97–1.18)	NA		
Rwanda	88	85.10 (79.24–90.97)	1.00	68	88.94 (84.24–93.63)	1.06 (0.92–1.23)	44	95.21 (89.95–100.00)	1.12 (0.93–1.35)	NA		

Note: Upper age limits for individual eligibility varied by country: 59 years of age for Lesotho and Zambia, 64 years of age for Cameroon, Cote d'Ivoire, Ethiopia, Kenya, Malawi, Namibia, Rwanda and Uganda, and no upper age limit in Eswatini, Tanzania and Zimbabwe.

^a^ Adjusted for sex, residence, employed, wealth quintile, education, marital status and partner HIV status‐self‐reported.

**Table 4:** Poisson regression models for antiretroviral therapy use among those aware of HIV‐positive status (second 90), Population‐based HIV Impact Assessments, 13 countries, 2015–2019

	Aged 50–54			Aged 55–59			Aged 60–64			Aged 65+		
Country	*n*	% (95% CI)	aPR^a^	*n*	% (95% CI)	aPR^a^	*N*	% (95% CI)	aPR^a^	*n*	% (95% CI)	aPR^a^
Malawi	126	96.29 (94.56–98.01)	1.00	85	94.47 (93.34–95.59)	1.01 (0.96–1.07)	57	94.5 (90.03–98.97)	0.99 (0.94–1.04)	NA		
Zambia	164	94.13 (91.05–97.21)	1.00	92	95.67 (93.06–98.28)	1.03 (0.95–1.11)	NA			NA		
Eswatini	189	91.85 (88.13–95.57)	1.00	131	93.31 (89.35–97.26)	1.01 (0.94–1.08)	109	98.79 (97.51–100.00)	1.06 (1.01–1.11)*	82	98.04 (95.31–100.00)	1.06 (1.00–1.11)
Tanzania	103	96.76 (95.83–97.69)	1.00	75	99.09 (97.58–100.00)	1.02 (0.98–1.05)	43	100.00 (100.00–100.00)	1.01 (0.96–1.05)	27	91.77 (77.97–100.00)	0.94 (0.66–1.32)
Zimbabwe	235	97.63 (95.90–99.35)	1.00	189	97.10 (95.42–98.77)	1.00 (0.96–1.03)	113	94.11 (90.28–97.95)	0.98 (0.93–1.03)	90	98.33 (95.99–100.00)	1.02 (0.97–1.07)
Uganda	105	93.62 (90.34–96.90)	1.00	66	96.35 (95.73–96.98)	1.03 (0.94–1.12)	52	94.04 (92.59–95.49)	1.01 (0.93–1.11)	NA		
Lesotho	253	95.43 (93.32–97.53)	1.00	222	97.94 (96.54–99.34)	1.01 (0.98–1.05)	NA			NA		
Cameroon	54	99.95 (99.83–100.00)	1.00	31	92.95 (91.73–94.17)	0.95 (0.89–1.02)	36	95.48 (94.55–96.41)	0.95 (0.91–0.99)*	NA		
Namibia	215	95.83 (92.99–98.67)	1.00	147	98.2 (96.43–99.96)	1.01 (0.97–1.05)	89	98.90 (96.71–100.00)	1.02 (0.97–1.06)	NA		
Cote d'Ivoire	24	95.39 (94.04–96.75)	1.00	20	96.45 (91.10–100.00)	1.01 (0.96–1.05)	11	100.00 (100.00–100.00)	1.11 (0.37–3.40)	NA		
Ethiopia	41	97.39 (96.76–98.03)	1.00	15	100.00 (100.00–100.00)	1.04 (0.94–1.16)	17	91.13 (88.96–93.3)	0.91 (0.74–1.12)	NA		
Kenya	129	96.67 (93.65–99.69)	1.00	85	97.83 (94.66–100.00)	1.02 (0.89–1.16)	62	100.00 (100.00–100.00)	1.04 (0.95–1.14)	NA		
Rwanda	87	99.21 (99.10–99.31)	1.00	68	100.00 (100.00–100.00)	1.01 (1.00–1.02)*	43	97.23 (96.81–97.65)	0.98 (0.96–1.00)*	NA		

^a^ Adjusted for sex, residence, employed, wealth quintile, education, marital status, partner HIV status‐self‐reported and the number of years since diagnosis.

* *p* < 0.05.

**Table 5:** Poisson regression models for viral load suppression among those on antireotroviral therapy (third 90), Population‐based HIV Impact Assessments, 13 countries, 2015–2019

	Aged 50–54			Aged 55–59			Aged 60–64			Aged 65+		
Country	*n*	% (95% CI)	aPR^a^	*n*	% (95% CI)	aPR^a^	*n*	% (95% CI)	aPR^a^	*n*	% (95% CI)	aPR^a^
Malawi	119	93.43 (90.02–96.84)	1.00	79	91.71 (86.25–97.18)	1.01 (0.89–1.15)	52	93.78 (90.32–97.25)	0.99 (0.88–1.12)	NA		
Zambia	154	93.49 (90.61–96.37)	1.00	91	99.39 (99.29–99.48)	1.07 (0.99–1.15)	NA			NA		
Eswatini	182	96.81 (94.61–99.02)	1.00	125	95.86 (92.55–99.18)	1.01 (0.95–1.07)	107	98.51 (96.43–100.00)	1.01 (0.98–1.05)	77	94.3 (89.04–99.55)	0.97 (0.89–1.05)
Tanzania	91	91.78 (89.08–94.49)	1.00	70	90.58 (81.37–99.78)	1.01 (0.90–1.15)	38	87.68 (81.39–93.98)	0.90 (0.72–1.11)	25	90.04 (84.80–95.28)	1.06 (0.97–1.16)
Zimbabwe	215	90.89 (87.67–94.11)	1.00	177	93.96 (90.87–97.05)	1.05 (0.98–1.12)	106	93.72 (88.1–99.35)	1.05 (0.96–1.14)	81	88.03 (81.05–95.01)	1.01 (0.89–1.14)
Uganda	93	88.63 (84.27–93.00)	1.00	58	86.75 (81.94–91.56)	1.06 (0.86–1.31)	39	77.02 (66.61–87.42)	0.94 (0.68–1.29)	NA		
Lesotho	233	91.97 (88.96–94.99)	1.00	211	95.03 (92.98–97.09)	1.01 (0.96–1.07)	NA			NA		
Cameroon	46	86.78 (78.44–95.11)	1.00	29	91.03 (89.34–92.71)	1.22 (0.69–2.18)	33	92.02 (84.51–99.54)	1.07 (0.82–1.39)	NA		
Namibia	197	93.29 (89.75–96.83)	1.00	137	93.92 (91.34–96.51)	0.99 (0.94–1.05)	85	96.73 (93.47–100.00)	1.03 (0.96–1.12)	NA		
Cote d'Ivoire	23	94.75 (92.55–96.94)	1.00	18	95.47 (95.12–95.81)	1.09 (0.49–2.45)	9	74.69 (55.14–94.24)	0.94 (0.72–1.23)	NA		
Ethiopia	38	92.56 (90.69–94.43)	1.00	14	98.07 (97.15–98.98)	1.01 (0.87–1.17)	15	86.20 (73.10–99.29)	1.10 (1.00–1.20)*	NA		
Kenya	120	93.01 (90.07–95.96)	1.00	80	94.61 (93.24–95.99)	1.02 (0.93–1.12)	60	97.00 (96.50–97.49)	1.03 (0.92–1.14)	NA		
Rwanda	81	92.52 (88.95–96.09)	1.00	63	91.37 (85.78–96.96)	1.01 (0.90–1.14)	38	87.36 (79.59–95.13)	0.98 (0.87–1.11)	NA		

^a^ Adjusted for sex, residence, employed, wealth quintile, education, marital status, partner HIV status‐self‐reported and the number of years since initiating ART.

* *p* < 0.05.

## 4 DISCUSSION

Overall, OPLWH have made more progress towards achievement of 90‐90‐90 UNAIDS targets than PLWH aged 15–49. Progress by OPLWH towards 90‐90‐90 UNAIDS targets was noted in all 13 countries included in this analysis, which suggests they are well positioned to reach the 95‐95‐95 targets by 2025 as well. In two countries, Eswatini and Namibia, OPLWH have achieved all three 90‐90‐90 targets. In the other 11 countries, OPLWH have all achieved one or both of the second and third 90‐90‐90 targets. However, achievement of the first 90, that is awareness of HIV‐positive status, continues to be a challenge [36, 37].

In the models we used, there was minimal variation demonstrated by age strata among OPLWH in the 13 countries. There was no variation in awareness by older age groups, three countries showed variation in treatment by older age groups—although not in a consistent direction, and one country showed higher VLS by an older age group. This lack of difference by age strata among OPLWH could indicate that those who have survived with HIV over the age of 50 are experienced with taking medications and older age does not diminish that fact.

Our findings are consistent with those from both a modelling study utilizing UNAIDS data and results from a systematic review and meta‐analysis of 92 studies, which showed heterogeneity of 90‐90‐90 progress across countries, sex and age groups [5, 9]. Both studies, which did not specifically focus on OPLWH, noted more progress towards 90‐90‐90 targets among OPLWH compared with younger age groups, but neither examined progress among OPLWH in the various age strata within that group [5, 9]. Our findings among PLHIV aged 15–49 years were similar to other research as we also found that women were more likely to achieve the 90‐90‐90 targets than men; however, we noted different 90‐90‐90 progress among men and women among OPLWH in our study, with men making more progress to achieve the first and third 90 targets than women, while second 90 achievement was similar [5, 9].

A key finding highlighted by our study is the gap noted in the achievement of the first 90, that is awareness of HIV‐positive status, among OPLWH [10, 37]. We found that 21.3% of all OPLWH were unaware of their HIV‐positive status before the surveys, and more than half (54%) of these unaware OPLWH had never previously been tested. Existing research suggests that older adults have less knowledge and understanding of HIV infection than younger age groups and are, therefore, less likely to seek testing [37]. Providers often do not consider older adults to be at risk for HIV so there is hesitation to discuss risk behaviours associated with HIV acquisition, such as inquiring into sexual activity and other risk behaviours, though older adults may have similar risks of HIV infection as younger adults [8, 10, 11, 38–40]. This provider bias combined with a lack of understanding of ongoing transmission risk for HIV infection in this population among both the providers and the older adults results in many missed opportunities for HIV testing, access to HIV prevention and initiation and retention in treatment services. Country HIV programmes could benefit from additional strategies to raise awareness among healthcare providers and increase health communication campaigns about testing and existing prevention interventions (such as knowledge of partner HIV status and the use of condoms, PrEP or treatment as prevention [U = U] for discordant couples) targeting older populations in SSA [41, 42].

Many OPLWH will wait to test for HIV until symptomatic which results in a delay in diagnosis of HIV and initiation of ART, resulting in poorer health outcomes [8, 38, 43]. In addition, age‐disparate relationships are associated with higher HIV acquisition among adolescent girls and young women [44]. As such, more effective case‐finding among older men may also have positive consequences on reducing onward transmission. Innovative testing strategies to reach older adults, such as establishing tailored testing initiatives for this age group, use of self‐testing and delivery of testing through community‐based services, have been shown to be useful to increase more frequent and earlier testing [5, 45]. In addition, OPLWH are managing other chronic health conditions and are at higher risk of developing age‐associated non‐communicable diseases, such as cardiovascular disease, neurocognitive disorders and fraility [46]. Older adults may be more likely to seek medical care for reasons other than HIV but provider‐initiated counselling and testing may be overlooked for this population because they are thought not to be at risk [47]. These findings suggest the need for further sensitization and training for health providers to elicit sexual and history of risk behaviours among older patients and to offer them HIV testing as a comprehensive strategy to identify all HIV‐positive individuals and combining with non‐HIV care management, as part of routine provider‐initiated counselling and testing [48, 49].

The finding that OPLWH were more likely to be aware of their HIV‐positive status than younger PLWH may be due to survivor bias; those who remained unaware of their HIV‐positive status longer were more likely to have died compared to those who became aware sooner, that is at a young age. Studies have shown that age is associated with adherence to ART and achievement of VLS [50]. One study that assessed mortality in SSA among HIV‐positive individuals on ART found that sex differences in all‐cause mortality and loss‐to‐follow‐up noted in younger people were also present among those ages 50–59, with older women on ART at greater risk of death as they age compared with men [51]. Additionally, there is a potential cohort effect for mortality among the OPLWH who have survived to this point compared with younger PLHIV [38, 52]. There was a global mortality peak from AIDS‐related deaths in 2005/2006, at a time when our OPLWH cohort was aged 35–50, and since 2010, there has been a 50% reduction in AIDS‐related deaths in Eastern and Southern Africa [53]. Additionally, PHIA surveys in many countries were conducted before test and treat protocols and treatment with dolutegravir was fully rolled out, potentially contributing to some countries not achieving the third 90 yet [54, 55, 56].

The study has several strengths. One key feature is that the PHIA surveys provided nationally representative data for all 13 countries, including HIV‐related biomarkers. Because the PHIA surveys are collected in the household, they capture information on those who may not be engaged in care and accessing health services, including older people who may be living with HIV and have yet to access treatment. The findings highlight the importance of expanding the age limits of all HIV surveys and surveillance efforts, to track progress among the growing population of OPLWH. The study also has some limitations. The surveys were all cross‐sectional in nature and only represent one time point. However, repeat PHIA surveys have been done in several countries, which will permit the assessment of trends among OPLWH over time in the future. Additionally, the upper age ranges varied across PHIA surveys: two countries did not have data for ages above 59, and eight countries did not have data for ages 65 and older. We examined results of 90‐90‐90 achievement by upper age limit in each country and did not observe any differences in 90‐90‐90 achievement between countries based on upper age limit; successful achievement was distributed across all countries.

## 5 CONCLUSIONS

In conclusion, finding gaps in the knowledge of HIV‐positive status among OPLWH in several African countries highlights the importance of focused HIV awareness and the need to expand HIV testing efforts for older adults, as it impacts the care and treatment cascade and reduces longevity and quality of life. At the same time, HIV programmes and providers could use these findings to support improved HIV counselling, education and screening of older adults on risk behaviours and offer them HIV prevention, testing and treatment services.

## COMPETING INTERESTS

None to declare for any authors.

## AUTHORS’ CONTRIBUTIONS

All authors have read and approved the manuscript. SMF conceptualized, drafted and edited the manuscript. CW and RB conceptualized and conducted analyses and edited the manuscript. AL, SD, DH, ANK and TGH conceptualized and edited the manuscript. RN, NW, ML, MA, JMJ, NK, IP, NM, SSN, AOA, EWN, FMA, SL, JW, PC, OM, BKM, PN, DA, AK, SB, FN, GM, CBN, JDS, EKD, LED, RM‐S, A‐CB, YG, FE, TN, LT, RFK, WK, JM, SB, EK, GR, AA, CW, SB, KS, HKP, KB and ACV reviewed and edited the manuscript. WME and JJ conceptualized, reviewed and edited the manuscript.

## FUNDING

The PHIAs were funded by the President's Emergency Plan for AIDS Relief through the US Centers for Disease Control and Prevention (CDC) under the terms of cooperative agreements #1U2GGH000994 and 5NU2GGH001226.

## DISCLAIMER

The findings and conclusions in this manuscript are those of the authors and do not necessarily represent the official position of the funding agencies.

## Data Availability

The data that support the findings of this study are available upon request from the PHIA project website: https://phia‐data.icap.columbia.edu/.
